# Conditional survival in multiple myeloma and impact of prognostic factors over time

**DOI:** 10.1038/s41408-023-00852-4

**Published:** 2023-05-15

**Authors:** Nadine H. Abdallah, Alexandra N. Smith, Susan Geyer, Moritz Binder, Patricia T. Greipp, Prashant Kapoor, Angela Dispenzieri, Morie A. Gertz, Linda B. Baughn, Martha Q. Lacy, Suzanne R. Hayman, Francis K. Buadi, David Dingli, Yi L. Hwa, Yi Lin, Taxiarchis Kourelis, Rahma Warsame, Robert A. Kyle, S. Vincent Rajkumar, Shaji K. Kumar

**Affiliations:** 1 Division of Hematology, Mayo Clinic, Rochester, MN USA; 2 Department of biostatistics, Mayo Clinic, Rochester, MN USA; 3 Department of Laboratory Medicine and Pathology, Mayo Clinic, Rochester, MN USA

**Keywords:** Epidemiology, Risk factors

## Abstract

Overall survival estimates from diagnosis are valuable for guiding treatment, but do not consider the years already survived. Conditional survival (CS) provides dynamic survival predictions over time. This study was conducted to estimate CS at 1–8 years from diagnosis and the impact of baseline prognostic factors on CS in multiple myeloma (MM) patients. This is a retrospective study including 2556 MM patients diagnosed between 2004 and 2019. CS (*t* | s) was defined as the probability of surviving *t* years given survival of *s* years. Median age was 64 years. Median follow-up was 6.2 years and median overall survival from diagnosis was 7.5 years. The 5-year CS estimates at *s* = 0, 1, 2, 3, and 5 years were 0.64, 0.61, 0.61, 0.61, and 0.58, respectively. On multivariate analysis, age ≥ 65 and proteasome inhibitor+immunomodulatory-based induction were associated with decreased survival and increased survival, respectively, retained at 5 years. The adverse impact of 1q gain/amplification, high-risk IgH translocation, and ISS-3 was significant at 1 and 3 years but not 5 years. Chromosome 17 abnormality was associated with decreased survival only at 1 year. Among MM patients, 5-year CS was stable at 1–5 years from diagnosis. The prognostic impact of high-risk cytogenetic factors decreased with additional years survived.

## Introduction

Multiple myeloma (MM) is a plasma cell disorder characterized by marked heterogeneity in terms of clinical, cytogenetic, and molecular disease characteristics which is reflected in varying prognosis for individual patients. Survival estimates from the time of diagnosis incorporating known disease- and host- specific risk factors are the basis for myeloma risk stratification systems [1, 2]. Although these survival estimates are important for predicting prognosis and guiding treatment selection for newly diagnosed patients, they are less pertinent for patients who have been on therapy as they do not take into account the number of years a person has already survived from diagnosis. Conditional survival (CS), defined as the probability that a person will survive an additional number of years if they have already survived “x” years, provides a dynamic and updated prediction of survival over the disease course which can guide disease monitoring strategies and prognostication. Such estimates are increasingly important as expansion of the treatment armamentarium has resulted in deeper responses and higher rates of complete remission translating to improved survival [3, 4], with a subset of patients achieving long-term survival approaching that expected for the age- and sex-matched general population [5]. CS estimates have been reported for both non-malignant [6] and malignant diseases, with the latter including a wide range of tumor types and stages [7]. Unlike other malignant conditions where CS improves as the number of years survived increases [8–10], several studies have suggested that CS in MM is stable [11, 12] or improves minimally over time [9, 13]. It is unclear whether this still holds true in the era of novel therapy and effective salvage therapies. Like traditional overall survival (OS) predictions from diagnosis, CS estimates at each time point can be refined by incorporating known prognostic risk factors available at that time. However, current prognostic factors in MM were established based on their impact on survival in newly diagnosed patients, and it is unclear whether these same risk factors retain their prognostic influence in those who have survived to a given time point. This study was conducted to estimate CS at (i.e., conditional on surviving to) 1 to 8 years from diagnosis in a large cohort of newly diagnosed patients with MM treated with contemporary regimens, and to evaluate the relative impact of prognostic factors from the time of diagnosis on CS estimates at different time points, with a focus on cytogenetic abnormalities.

## Methods

### Patient population

A total of 2556 patients diagnosed with MM between February 15th, 2004, and June 20th, 2019, were included. Patients were identified from a preexisting database at Mayo Clinic in Rochester, MN. Additional clinical, laboratory, and cytogenetic data were obtained by review of electronic medical records. All included patients authorized the use of their medical record information for research purposes. The study was approved by the Mayo Clinic Institutional Review Board.

### Conditional survival

Conditional survival CS (*t* | *s*) was defined as the probability of surviving an additional number of years (*t*), conditional on survival of (*s*) years. We estimated CS (*t* | *s*) at different landmark points *s* where *s* = 0, 1, 2, 3, 5, and 8 corresponding to time from diagnosis in years. Median CS was estimated for patients who were alive at each timepoint. We also plotted the 5-year CS (5|*s*), the probability of surviving 5 years given that a person has already survived s years, as a function of each prediction time *s* (*s* = 1, 2, 3, 5) for the entire cohort, and in different groups stratified by age ( < 65 and ≥ 65 years), induction treatment, R-ISS stage (III vs. I/II), and cytogenetic risk profile. To mitigate the potential influence of less follow-up in those diagnosed after 2013, we also estimated 3-year CS separately for patients diagnosed before and after 2013. Survival was defined as the time from *s* until death from any cause. Patients who were still alive at their last follow-up were censored. CS estimates were calculated using the conditional Kaplan-Meier method and compared between groups using the Log-rank test [14]. All statistical analyses were performed using the R version 4.1.2 statistical software. Two-sided *p*-values < 0.05 were considered statistically significant.

### Univariate and multivariate analysis

We evaluated the impact of patient- and disease- related characteristics on CS at different timepoints *s* (*s* = 0, 1, 2, 3, 5) using conditional Cox proportional hazards models, expressed as hazard ratios (HR) (and 95% confidence intervals [CI]). Multivariate models were constructed at each timepoint *s* including variables that were associated with survival on univariate analysis with a 2-sided *p*-value of < 0.05. On the final multivariate model, 2-sided *p*-values < 0.05 were considered statistically significant. All prognostic variables were extracted from the time of diagnosis and included clinical, laboratory, and cytogenetic variables. Since analyses are conditional on survival to specific time points and are based on baseline factors, these analyses utilized conditional survival (CS) analyses instead of landmark analyses.

### Cytogenetic data

All included patients had cytogenetic analysis performed by FISH within 1 year from diagnosis and within 6 months of starting first-line treatment. The method for FISH testing has been previously described [15]. Briefly, unsorted bone marrow plasma cells were identified by cytoplasmic immunoglobulin staining, and FISH was performed using a panel that included the following enumeration probes: 3 (D3Z1), 7 (D7Z1), 9 (D9Z1), and 15 (D15Z4) centromeres, -13q14 (RB1/LAMP1), -13q (RB1/LAMP1), -17p13.1 (TP53/D17Z1), and -17 (TP53/D17Z1) (Abbott Molecular, Des Plaines, IL). A break apart probe targeting IgH was used to detect an IgH rearrangement, and dual-color, dual-fusion probes t(11;14) CCND1/IgH were used to detect the t(11;14)(q13;q32) translocation (Abbott Molecular). Reflex testing using dual-color, dual-fusion probes was done to identify other partners for the IgH translocation if an IgH rearrangement other than t(11;14) was detected: t(4;14)(p16.3;q32) FGFR3/IgH, t(14;16)(q32;q23) IgH/MAF, t(14;20)(q32;q12) IgH/MAFB, and t(6;14)(p21;q32) CCND3/IgH (all from Abbott Molecular). 1q gain and MYC rearrangements were determined using the 1q/1p (1q22/TP73) (in house, custom developed) and the 8q24.1 break apart probes (Abbott Molecular), respectively [16, 17]. The t(4;14), t(14;16), and t(14;20) translocations were considered high-risk [18]. A high-risk cytogenetic abnormality was defined by the presence of any of: a high-risk IgH translocation, a chromosome 17 abnormality (del17p or monosomy of chromosome 17, and/or 1q gain (gain of 1 or more copies of 1q22). We defined double- and triple hit disease by the presence of a high-risk IgH translocation (primary high-risk cytogenetic abnormality) in addition to 1 (double-hit) or 2 (triple-hit) of the following abnormalities: chromosome 17 abnormality and/or 1q gain (secondary cytogenic abnormalities) [19].

### Clinical and laboratory data

Hypercalcemia was defined as serum calcium ≥ 11 mg/dL, renal failure was defined as serum creatinine ≥ 2 mg/dL, and thrombocytopenia was defined as platelets < 150,000 /microliter of blood. High LDH was defined as any value above the upper limit of normal based on the reference range of the laboratory at which the test was performed (222 IU/L in Mayo Clinic Laboratories). Early transplant was defined as autologous stem cell transplantation (ASCT) performed within 12 months from diagnosis. Patients were grouped into three disease stages using the ISS [1] based on β2-microglobulin and albumin levels, and R-ISS [2] based on ISS stage, normal vs. elevated LDH level, and presence or absence of high-risk cytogenetics. For R-ISS staging, high-risk cytogenetics was defined by the presence of a high-risk IgH translocation and/or del(17p) [2].

## Results

### Baseline characteristics

Our analysis cohort consisted of 2556 patients diagnosed with MM between February 15th, 2004, and June 20th, 2019, with risk factor data available at diagnosis. The median age was 64 (range: 22–96) years and 49% were ≥ 65 years; 62% were male. At the time of diagnosis, 25% had ISS I, 39% had ISS II, and 35% had ISS III disease. Among all patients, 51% had at least 1 high-risk abnormality, including 9% and 1% with double- and triple- hit MM, respectively. The first-line induction regimens were proteasome inhibitor (PI)-based (31%), immunomodulatory drug-(IMiD)-based (31%), PI+IMiD-based (34%) and other (5%); 46% underwent early transplant. The baseline characteristics of this cohort have been previously described [20]. The median follow-up in the entire cohort was 6.2 (95%CI: 5.9–6.5) years. The median OS from diagnosis for the entire cohort was 7.5 (95%CI: 7.0–8.1) years and the estimated 5- and 10-year OS rates from diagnosis were 64% and 37%, respectively. Among all patients, 156 died within 1 year from diagnosis, and 64 had less than 1 year follow up but were still alive at last follow-up; 2336, 1573, 977, and 429 patients were alive at 1, 3, 5, and 8 years from diagnosis. The characteristics of patients who were alive at different timepoints are shown in Table 1a, b. Conditioning on survival 1 to 8 years from diagnosis, the percentage of patients with known high-risk factors at diagnosis decreased with increasing time from diagnosis: age ≥ 65 years (47% to 32%), ISS III (34% to 23%), R-ISS III (16% to 8%), hypercalcemia (10% to 5%), renal failure (15% to 10%), high LDH (16% to 11%) and thrombocytopenia (18% to 13%). There was an increase in the proportion of patients who had normal FISH at diagnosis (6% to 11%), but a decrease in the proportion who had a high-risk IgH translocation (14% to 7%), 1q gain (30% to 14%), MYC abnormality (9% to 5%), deletion 13q (10% to 7%), monosomy 13 (36% to 29%), and chromosome 17 abnormality (13% to 7%). The proportion of patients with double- or triple- hit cytogenetic abnormality also decreased with increasing years survived (16% to 5%). The proportion of patients who received IMiD-based induction increased at increasing CS time points (31% at *s* = 1 to 48% at *s* = 5 and 66% at *s* = 8); the proportion of those who received PI-based induction was stable at *s* = 1, 3, and 5 years (30%, 32% and 29%, respectively), but decreased to 15% at 8 years. The proportion of patients who received PI+IMiD-based induction decreased progressively (35% at *s* = 1, 18% at *s* = 5, and 13% at *s* = 8 years). The proportion of patients who underwent early transplant was relatively stable (50% at *s* = 1 and 49% at *s* = 8). The mean plasma cell percentage at diagnosis was also similar in patients alive at increasing timepoints (50% at *s* = 1 and 47% at *s* = 8).

**Table 1:** Characteristics of patients surviving a minimum of 1, 3, 5, and 8 years from diagnosis.

	Died < 1 year from diagnosis (*N* = 156)	OS > 1 year from diagnosis (*N* = 2336)	OS > 3 years from diagnosis (*N* = 1573)	OS > 5 years from diagnosis (*N* = 977)	OS > 8 years from diagnosis (*N* = 429)
**a:** Clinical and laboratory characteristics of patients surviving a minimum of 1, 3, 5, and 8 years from diagnosis.					
Age(years) Mean (SD)	70.5 (11.3)	63.2 (10.2)	62.4 (10.1)	61.5 (9.9)	59.5 (9.9)
Age ≥ 65 years	108 (69.2%)	1105 (47.3%)	687 (43.7%)	398 (40.7%)	139 (32.4%)
Sex (Male)	90 (57.7%)	1458 (62.4%)	957 (60.8%)	581 (59.5%)	247 (57.6%)
ISS					
Missing (*N*)	32	498	348	231	115
ISS 1	11 (8.9%)	483 (26.3%)	347 (28.3%)	228 (30.6%)	106 (33.8%)
ISS 2	35 (28.2%)	738 (40.2%)	506 (41.3%)	318 (42.6%)	137 (43.6%)
ISS 3	78 (62.9%)	617 (33.6%)	372 (30.4%)	200 (26.8%)	71 (22.6%)
R-ISS					
Missing (*N*)	46	686	480	306	144
R-ISS 1	7 (6.4%)	290 (17.6%)	217 (19.9%)	153 (22.8%)	78 (27.4%)
R-ISS 2	57 (51.8%)	1103 (66.8%)	741 (67.8%)	451 (67.2%)	184 (64.6%)
R-ISS 3	46 (41.8%)	257 (15.6%)	135 (12.4%)	67 (10.0%)	23 (8.1%)
BMPC% Mean (SD)	55.7 (30.2)	50.0 (25.9)	49.0 (25.6)	47.1 (25.3)	46.9 (25.7)
Missing (*N*)	14	231	156	114	61
Calcium ≥ 11 mg/dL	23 (18.5%)	187 (10.2%)	98 (8.0%)	48 (6.5%)	15 (5.0%)
Missing (*N*)	32	494	344	238	131
Creatinine ≥ 2 mg/dL	30 (24.4%)	281 (15.0%)	169 (13.3%)	78 (10.2%)	30 (9.8%)
Missing (*N*)	33	465	299	209	124
Albumin < 3.5 g/dL	89 (70.1%)	822 (46.3%)	520 (44.7%)	300 (42.9%)	132 (43.4%)
Missing (*N*)	29	561	409	277	125
B2M > 5.5 mcg/mL	77 (61.1%)	609 (30.4%)	371 (27.5%)	199 (23.5%)	71 (19.3%)
Missing (*N*)	30	330	223	131	61
Elevated LDH	39 (36.4%)	255 (15.5%)	156 (14.2%)	85 (12.5%)	34 (11.1%)
Missing (*N*)	49	688	476	297	124
Platelets < 150/mcL	42 (37.8%)	215 (18.1%)	136 (16.5%)	82 (15.1%)	29 (12.9%)
Missing (*N*)	45	1146	750	435	204
**b:** Cytogenetic characteristics and treatment regimens of patients surviving a minimum of 1, 3, 5 and 8 years from diagnosis.					
Normal FISH	7 (4.5%)	128 (5.5%)	100 (6.4%)	69 (7.1%)	48 (11.2%)
HR IgH trans	45 (29.0%)	321 (14.0%)	185 (12.0%)	99 (10.3%)	28 (6.6%)
Missing (*N*)	1	42	29	15	2
HR IgH trans/ 1q gain/ Ch17 Abn	92 (75.4%)	892 (49.0%)	510 (44.1%)	250 (38.2%)	78 (29.7%)
Missing (*N*)	34	514	417	323	166
Double or Triple Hit	31 (50.8%)	174 (15.8%)	84 (11.5%)	33 (7.6%)	9 (4.6%)
Missing (*N*)	95	1232	843	540	235
1q gain	58 (52.7%)	514 (29.7%)	276 (25.5%)	109 (18.2%)	33 (13.8%)
Missing (*N*)	46	608	490	377	190
Myc Abn	14 (13.6%)	145 (8.6%)	83 (7.9%)	45 (7.7%)	11 (4.7%)
Missing (*N*)	53	642	517	393	194
13q del	16 (10.3%)	233 (10.2%)	137 (8.9%)	88 (9.1%)	30 (7.1%)
Missing (N)	0	43	30	11	4
Monosomy 13	75 (48.1%)	830 (36.2%)	539 (34.9%)	316 (32.7%)	124 (29.1%)
Missing (*N*)	0	42	30	12	3
Ch17 Abn	36 (23.1%)	288 (12.6%)	158 (10.3%)	86 (8.9%)	31 (7.3%)
Missing (*N*)	0	56	38	16	4
1st line Treatment					
Missing (*N*)	29	140	70	43	20
IMiD	32 (25.2%)	683 (31.1%)	578 (38.5%)	446 (47.8%)	269 (65.8%)
PI	51 (40.2%)	667 (30.4%)	474 (31.5%)	275 (29.4%)	63 (15.4%)
PI+IMiD	25 (19.7%)	758 (34.5%)	388 (25.8%)	167 (17.9%)	51 (12.5%)
Other	19 (15.0%)	88 (4.0%)	63 (4.2%)	46 (4.9%)	26 (6.4%)
SCT	21 (13.5%)	1376 (58.9%)	1023 (65.0%)	667 (68.3%)	288 (67.1%)
Early SCT	21 (13.5%)	1161 (49.7%)	834 (53.0%)	530 (54.2%)	209 (48.7%)

*B2M* Beta2microglobulin, *BMPCs* Bone marrow plasma cells, *ISS* International staging system, *LDH* Lactate dehydrogenase, *N* number, *OS* Overall survival, *R-ISS* Revised international staging system, *SD* Standard deviation. *Abn* Abnormality, *Ch17* Chromosome 17, *Del* Deletion, *FISH* Fluorescence In Situ Hybridization, *HR* High-risk, *IMiD* Immunomodulatory drug, *N* number, *OS* Overall survival, *PI* Proteasome inhibitor, *SCT* Stem cell transplant, *trans* translocation.

### Conditional survival

**Fig. 1:**
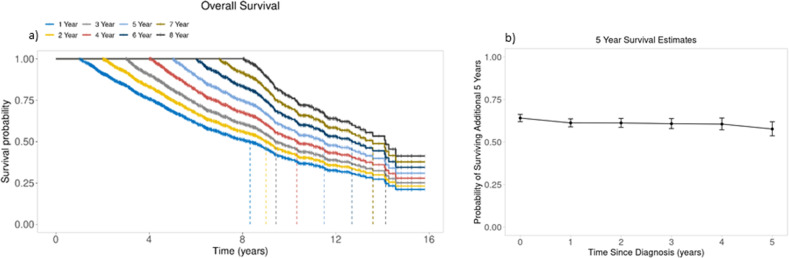
Conditional survival in the entire cohort. **a** Conditional survival curves at 1 to 8 years from diagnosis. **b** 5-year conditional survival at diagnosis and 1 to 5 years from diagnosis.

**Fig. 2:**
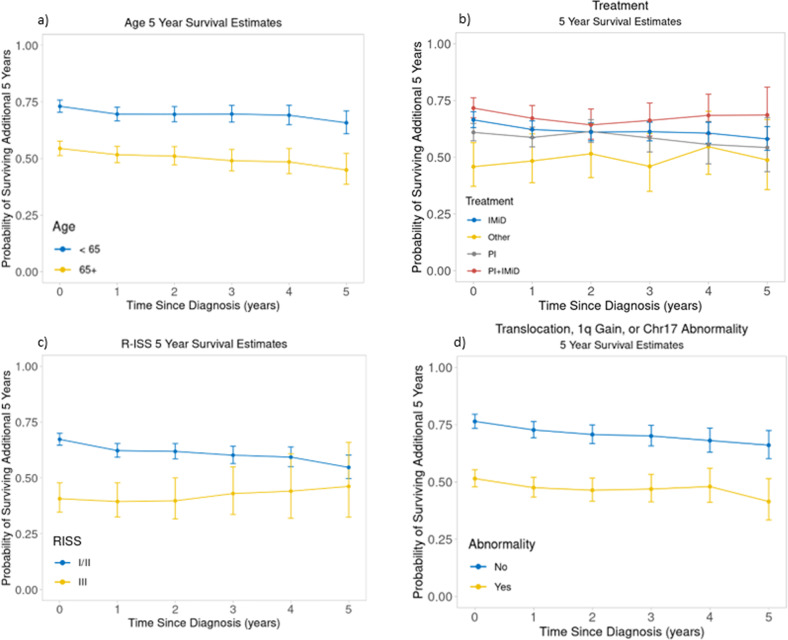
5-year conditional survival in different groups. 5-year conditional survival at diagnosis and 1 to 5 years among patients stratified by (**a**) age, (**b**) induction treatment, (**c**) R-ISS, and (**d**) presence of high-risk cytogenetic abnormality.

**Fig. 3:**
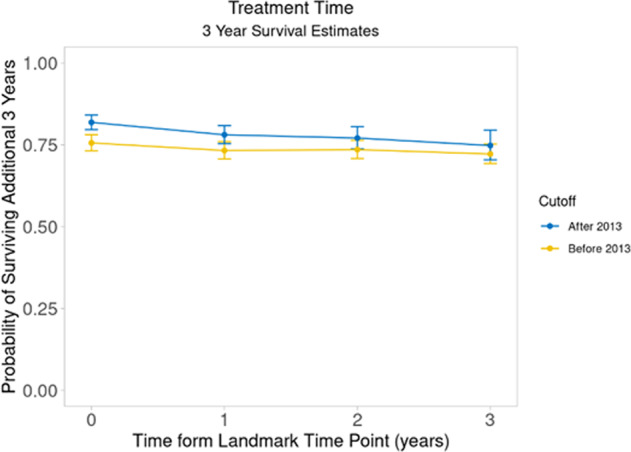
3-year conditional survival by diagnosis period. 3-year conditional survival at diagnosis, and at 1 to 3 years from diagnosis among patients diagnosed before and after 2013.

Median CS estimates at *s* = 0, 1, 2, 3, 4, 5, and 8 years from diagnosis were 7.5 (95%CI: 7.0–8.1), 8.3 (95%CI: 7.6–8.8), 9.0 (95%CI: 8.6–9.4), 9.4 (95%CI: 9.2–10.1), 10.3 (95%CI: 9.7–11.5), 11.5 (95%CI: 10.4–12.8) and 14.1 (95%CI: 13.3-not reached [NR]) years (Fig. 1a). For the entire cohort, 5-year CS estimates at *s* = 0, 1, 2, 3, 4, and 5 years from diagnosis were 0.64 (95%CI: 0.62–0.66), 0.61 (95%CI: 0.59–0.64), 0.61 (95%CI: 0.59–0.64), 0.61 (95%CI: 0.58–0.64), 0.61 (95%CI: 0.57–0.64) and 0.58 (95%CI: 0.54–0.62), respectively (Fig. 1b). The 5-year CS probability estimates at each time point *s* stratified by age, induction treatment, R-ISS stage, and presence or absence of a high-risk cytogenetic abnormality are shown in Fig. 2a–d. We then estimated 3-year CS separately by diagnosis period (before vs. after 2013). Among all 2556 patients, 1170 were diagnosed with MM before 2013, and 1386 were diagnosed after. We found the following differences in induction regimen between the 2 time periods (i.e., before vs. after 2013): IMiD-based in 56% vs. 9%, PI-based in 23% vs. 3%, PI+IMiD-based in 13% vs. 53%, and other in 9% vs. 1%, respectively. The median follow-up for those diagnosed prior to 2013 was 10.0 (95% CI: 9.7–10.4) years, whereas the median follow-up for those diagnosed after 2013 was 3.7 (95% CI: 3.5–3.9) years. Among patients diagnosed before 2013, 3-year CS at *s* = 0, 1, 2, and 3 years was 0.76 (95%CI: 0.73–0.78), 0.73 (95%CI: 0.71–0.76), 0.74 (95%CI: 0.71–0.76], and 0.72 (95%CI: 0.69–0.75), respectively. Among patients diagnosed after 2013, median conditional 3-year CS at *s* = 0, 1, 2, and 3 years was 0.82 (95%CI: 0.8–0.84), 0.78 (95%CI: 0.75–0.81), 0.77 (95%CI: 0.74–0.81), and 0.75 (95%CI: 0.7–0.79), respectively (Fig. 3).

### Impact of baseline prognostic factors over time

On univariate analysis, age ≥65 years was associated with an increased risk of death with a stable impact over time. Thrombocytopenia was associated with an increased risk of death at all timepoints *s* with only a small decrement in HR over time. The adverse impact of advanced ISS and R-ISS on CS decreased steadily with increased time survived. Renal failure was associated with a significantly increased risk of death from time of diagnosis and in those who survived to 1 and 3 years, but this was not statistically significant in those who survived to 5 years. Hypercalcemia was associated with increased risk of death at 1 and 2 years but this was no longer statistically significant at 3 and 5 years. The prognostic impact of LDH was not significant at 2 and 5 years. The presence of a high-risk IgH translocation at diagnosis remained significantly associated with decreased survival at all CS time points with a small decrement in the HR with time (HR: 1.96 and 1.68 at *s* = 0 and *s* = 5 years, respectively). Chromosome 17 abnormality was associated with an increased risk of death from *s* = 0 to *s* = 3 years, although the magnitude of effect decreased with additional years survived. However, in those surviving 5 years, it was no longer significantly associated with decreased survival. Monosomy 13 was associated with a modestly increased hazard for death that was stable from *s* = 0 to *s* = 3 years but it was no longer prognostic in those who survived to 5 years. MYC abnormality and 1q gain were both associated with an increased hazard for death which was maintained until *s* = 5 years. Interestingly, the CS HR for MYC abnormality was highest at *s* = 5 years (HR: 2.19) compared to earlier timepoints. Early transplant and PI+IMiD-based induction were both associated with improved survival which was maintained until *s* = 5 years (Table 2).

**Table 2:** Univariate analysis for CS at diagnosis and 1, 2, 3, and 5 years from diagnosis.

Variable	Survival from diagnosis	CS – year 1	CS – year 2	CS – year 3	CS – year 5
	HR0 (95%CI)	HR1 (95%CI)	HR2 (95%CI)	HR3 (95%CI)	HR5 (95%CI)
Age ≥ 65 years	1.95 (1.73–2.21)	1.89 (1.65–2.15)	1.86 (1.6–2.16)	1.88 (1.6–2.22)	1.91 (1.54–2.39)
Calcium ≥ 11 mg/dL	1.72 (1.40–2.12)	1.67 (1.33–2.11)	1.39 (1.04–1.86)	1.3 (0.92–1.83)	1.12 (0.65–1.92)
Creatinine ≥ 2 mg/dL	1.58 (1.33–1.89)	1.54 (1.27–1.88)	1.37 (1.08–1.74)	1.46 (1.12–1.91)	1.32 (0.89–1.96)
Platelets < 150/mcL	1.83 (1.54–2.19)	1.68 (1.37–2.05)	1.62 (1.28–2.04)	1.62 (1.25–2.11)	1.8 (1.26–2.57)
ISS III (vs I/II)	1.93 (1.69–2.21)	1.74 (1.50–2.02)	1.67 (1.41–1.97)	1.64 (1.36–1.98)	1.45 (1.11–1.90)
R-ISS III (vs I/II)	2.2 (1.86–2.61)	1.95 (1.61–2.37)	1.82 (1.44–2.29)	1.77 (1.36–2.32)	1.47 (0.99–2.20)
Early SCT	0.54 (0.48–0.61)	0.63 (0.55–0.71)	0.64 (0.55–0.74)	0.67 (0.57–0.79)	0.77 (0.61–0.96)
PI + IMiD induction	0.68 (0.58–0.81)	0.46 (0.39–0.55)	0.49 (0.4–0.61)	0.55 (0.4–0.74)	0.35 (0.18–0.67)
High LDH	1.62 (1.35–1.94)	1.4 (1.14–1.72)	1.21 (0.94–1.55)	1.33 (1.02–1.75)	1.1 (0.73–1.64)
Ch17 Abn	1.95 (1.66–2.29)	1.93 (1.62–2.30)	1.69 (1.36–2.09)	1.62 (1.26–2.07)	1.26 (0.85–1.85)
High-risk IgH trans	1.96 (1.68–2.28)	1.87 (1.58–2.21)	1.83 (1.50–2.23)	1.77 (1.41–2.22)	1.68 (1.21–2.34)
High-risk IgH trans/ Ch17 Abn/1q gain	2.35 (2.03–2.72)	2.25 (1.92–2.63)	2.23 (1.86–2.67)	2.09 (1.71–2.56)	1.88 (1.42–2.47)
Monosomy 13	1.40 (1.24–1.59)	1.37 (1.2–1.57)	1.33 (1.15–1.55)	1.32 (1.12–1.57)	1.14 (0.9–1.44)
MYC Abn	1.51 (1.18–1.93)	1.48 (1.13–1.94)	1.7 (1.26–2.29)	1.58 (1.10–2.27)	2.19 (1.37–3.50)
1q gain	1.84 (1.57–2.15)	1.71 (1.43–2.04)	1.92 (1.57–2.35)	1.87 (1.47–2.37)	1.97 (1.40–2.75)

*HR* Hazard ratio, *CS* Conditional survival, *CI* Confidence interval, *ISS* International staging system, *R-ISS* Revised international staging system, *SCT* Stem cell transplant, *PI* Proteasome inhibitor, *IMiD* Immunomodulatory drug, *LDH* Lactate dehydrogenase, *Abn* Abnormality, *trans* translocation, *Ch17* Chromosome 17.

On multivariate CS analysis, age ≥ 65 years (HR: 1.60), calcium ≥ 11 mg/dL (HR: 1.59), ISS III (HR: 1.74), chromosome 17 abnormality (HR: 1.53), high-risk IgH translocation (HR: 1.90), and 1q gain (HR: 1.34) were all significantly associated with decreased survival at diagnosis, while early transplant (HR: 0.57), and PI+IMiD-based induction (HR: 0.59) were associated with improved survival. High LDH and monosomy 13 were not significantly associated with survival at diagnosis. The prognostic impact of age ≥ 65, early transplant, and PI+IMiD induction on CS was maintained among survivors at 1, 2, 3, and 5 years from diagnosis. ISS III was associated with decreased CS at 1, 2, and 3 years, but this was not statistically significant in those who survived to 5 years. Similarly, the adverse prognostic impact of high-risk IgH translocation and 1q gain was no longer statistically significant in those surviving 5 years from diagnosis. The presence of chromosome 17 abnormality was associated with decreased CS at 1 year, but this was no longer statistically significant in those surviving to 2, 3 or 5 years from diagnosis (Table 3) (Supplemental Fig. 1).

**Table 3:** Multivariate analysis for CS at diagnosis and 1, 2, 3, and 5 years from diagnosis.

Variable	Survival from diagnosis	CS – year 1	CS – year 2	CS – year 3	CS – year 5
	HR0 (95%CI)	HR1 (95%CI)	HR2 (95%CI)	HR3 (95%CI)	HR5 (95%CI)
Age ≥ 65 years	1.60 (1.3–1.97)	1.57 (1.26–1.96)	1.55 (1.21–2.00)	1.48 (1.11–1.96)	1.95 (1.34–2.82)
Calcium ≥ 11 mg/dL	1.59 (1.17–2.16)	1.63 (1.16–2.29)	1.16 (0.73–1.85)	1.05 (0.59–1.86)	1.05 (0.46–2.41)
ISS III (vs I/II)	1.74 (1.42–2.14)	1.75 (1.4–2.19)	1.76 (1.36–2.28)	1.80 (1.34–2.41)	1.52 (0.99–2.32)
Early SCT	0.57 (0.46–0.70)	0.64 (0.51–0.80)	0.65 (0.50–0.83)	0.61 (0.46–0.81)	0.69 (0.48–1.00)
PI + IMiD induction	0.59 (0.46–0.76)	0.63 (0.48–0.82)	0.67 (0.48–0.92)	0.66 (0.45–0.98)	0.53 (0.29–0.98)
High LDH	1.29 (1.00–1.66)	1.15 (0.86–1.52)	0.96 (0.67–1.38)	1.13 (0.76–1.68)	0.89 (0.48–1.63)
Ch17 Abn	1.53 (1.19–1.98)	1.43 (1.07–1.90)	1.3 (0.92–1.82)	1.32 (0.89–1.95)	1.16 (0.64–2.09)
High-risk IgH trans	1.90 (1.48–2.45)	1.97 (1.49–2.61)	1.81 (1.29–2.55)	1.55 (1.03–2.34)	1.51 (0.80–2.85)
Monosomy 13	1.13 (0.92–1.37)	1.09 (0.88–1.36)	1.05 (0.82–1.34)	1.05 (0.79–1.39)	0.86 (0.59–1.25)
1q gain	1.34 (1.08–1.67)	1.31 (1.04–1.66)	1.51 (1.16–1.97)	1.50 (1.1–2.05)	1.38 (0.89–2.13)

*HR* Hazard ratio, *CS* Conditional survival, *CI* Confidence interval, *ISS* international staging system, *R-ISS* Revised international staging system, *SCT* Stem cell transplant, *PI* proteasome inhibitor, *IMiD* Immunomodulatory drug, *LDH* Lactate dehydrogenase, *Abn* Abnormality, *trans* Translocation, *Ch17* Chromosome 17.

## Discussion

Conditional survival (CS) has been reported for a wide range of solid and hematologic malignancies [7, 9, 10, 21], and in general has provided more favorable estimates of survival with additional years survived compared to OS estimates from diagnosis, with survival approaching that of the age-matched population for some malignancies [7, 9, 10, 21, 22]. CS estimates in MM have not been as optimistic compared to other hematologic malignancies, with studies reporting either stable CS or minimal improvement with time [8, 10, 11, 13, 23]. In a population-based Japanese study including patients diagnosed from 1993 to 2006, 5-year CS in MM patients (4914 patients) improved minimally from 33.7% to 44.9% at 1 to 5 years from diagnosis, respectively [8]. In another Canadian population-based study including patients diagnosed up to year 2006, there was improvement in 5-year conditional relative survival ratio (RSR) in patients with MM from 37% to 60% at 5 years, but this was less pronounced compared to other hematologic malignancies where conditional RSR approached 90% at 5 years (85% in non-Hodgkin’s lymphoma, 90% in leukemias other than chronic lymphocytic leukemia, and 95% in Hodgkin’s lymphoma) [10]. A more recent study by the German study group using data from 815 MM patients diagnosed between years 1997 and 2011 reported stable 5-year CS of approximately 50% from diagnosis to 5 years [11, 12]. Similarly, stable CS has been reported post stem cell transplantation even among patients with sustained CR [13, 23]. With improvement in survival of MM patients reflecting novel agents use, the question of cure in a subset of patients has been raised [24]. Similar to results from previous studies [11], our study, showed that as we condition on survival further out from diagnosis, patients with high-risk factors constitute a smaller percentage of those who have survived to the given time point of interest. The observation that the proportion of patients who received IMiD-based induction increased while the proportion of those who received PI- and PI+IMiD-based induction decreased with increased time survived, can be explained by the fact that the PI- and PI+IMiD-based regimens were more prevalent in those diagnosed in more recent years. Thus, looking at 8 year estimates we may not have sufficient follow-up to capture these more recently treated patients. Despite selection for more favorable patient- and disease-related risk features over time, 5-year CS was stable in patients surviving 1 to 5 years from diagnosis (61% to 58%, respectively) in our study, which spans a more recent period compared to previous studies (diagnosis between 2004 and 2019). CS was also stable to minimally changed among patients stratified by induction treatment, age, R-ISS stage, and cytogenetic risk profile. Survival differences between patients age < 65 and ≥ 65 years and those with vs. without a high-risk cytogenetic abnormality persisted over time in survivors, while the survival gap decreased among patients who survived 5 years from diagnosis for the different treatment groups and between patients with R-ISS stages I/II vs III. Our results, in conjunction with results from previous studies, suggest that despite improvement in outcomes, MM patients continue to have excess mortality even after surviving 5 years from diagnosis; this can be attributed to late recurrences, secondary malignancies, and late treatment-related toxicities among other causes. In a German study by Lehners et al. including 865 patients who underwent upfront ASCT from 1993 to 2014, long-term follow up beyond 5 years from ASCT did not identify a minimal time point predicting long-term survival. However, only 42% of patients received novel-based induction in this study, and this subset had shorter follow-up [13]. In another study by Ravi et al., young patients ( < 50 years) with MM had excess mortality even at 36 months from diagnosis compared to the age- and sex-matched general US population, with a standardized mortality ratio of 20.7. This was in contrast to young patients with diffuse large B cell lymphoma or Hodgkin’s lymphoma where survival was similar to the matched general population, suggesting cure [25].

In this study we also assessed the impact of known baseline prognostic variables among patients able to survive to different time points; on multivariate analysis, the adverse prognostic impact of older age at diagnosis ( ≥ 65 years) persisted even among those surviving to 5 years from diagnosis, while the impact of advanced ISS stage, high-risk IgH translocation, and 1q gain on survival was no longer statistically significant in patients surviving 5 years from diagnosis. The presence of a chromosome 17 abnormality was associated with decreased CS only at 1 year. These results suggest that risk stratification based on adverse cytogenetic abnormalities from the time of diagnosis may not be applicable for patients as they survive further out from diagnosis. On the other hand, the favorable impact of early transplant and PI+IMiD induction on survival was maintained even in patients who survived 5 years from diagnosis. Similar findings were observed in the study by Schinke et al. where disease stage and unfavorable cytogenetics, defined as any of high-risk IgH translocation, del(17p), hypodiploidy, MYC abnormality and chromosome 1 abnormality, were no longer prognostic after 5 years [11]. The lack of prognostic impact of high-risk cytogenetics at 5 years in their study and ours may also be related, at least partly, to the small sample size of patients surviving 5 years. Schinke et al. also used select prognostic variables (Karnofsky performance status, creatinine, and hemoglobin) to demonstrate that updated values at each landmark point have better predictive ability compared to the corresponding values from diagnosis [11]. Further studies evaluating updated clinical, laboratory and cytogenetic factors at each landmark point are needed to better define factors with prognostic impact among MM survivors.

The strengths of our study include a large sample size, use of novel-based induction in > 90% of patients, long follow-up, and availability of complete cytogenetic data allowing the evaluation of the impact of individual abnormalities over time. The limitations of this study are those inherent to real-world studies including missing data for some variables, a heterogenous population with variable follow-up, and long study period over which treatments have evolved. In addition, our study did not include data on treatment response or cytogenetic data at different timepoints, and thus the effect of treatment on the burden of the high-risk plasma cell clone over time could not be assessed.

In conclusion, this study shows that despite improvement in treatment strategies over time, MM continues to be associated with excess mortality, with no significant improvement in survival with additional years survived. Longer follow-up is needed to identify whether there is a timepoint beyond which survival plateaus, and to characterize the subset of patients achieving long-term remission. In addition, prognostic systems beyond the diagnosis period are needed to refine survival estimates in long-term survivors.

## Supplementary information

Supplementary Materials

## Data Availability

The datasets generated and analyzed during the current study are available from the corresponding author on reasonable request.
