# Supplementary material for: Conditional survival in multiple myeloma and impact of prognostic factors over time
Source: Blood Cancer J. 2023 May 15;13(1):78. doi: 10.1038/s41408-023-00852-4 (PMC10185675; doi:10.1038/s41408-023-00852-4)
Supplement: Supplementary file 1 — Supplementary Materials [file 41408_2023_852_MOESM1_ESM.pdf]

Supplemental Figure 1:

a)

1 Year Conditional Survival

Variable	P-value	HR [95% CI]
Age 65+	5.92e-05	1.57 [1.26-1.96]
Calcium 11+	0.005	1.63 [1.16-2.29]
LDH > 222	0.350	1.15 [0.86-1.52]
Early ASCT	8.82e-05	0.64 [0.51-0.8]
First-Line PI+IMID	7.85e-04	0.63 [0.48-0.82]
Ch17 Abnormality	0.015	1.43 [1.07-1.9]
ISS Stage III	7.60e-07	1.75 [1.4-2.19]
1q Gain	0.025	1.31 [1.04-1.66]
Monosomy 13	0.416	1.09 [0.88-1.36]
HR Translocation	2.26e-06	1.97 [1.49-2.61]
**Summary**		**1.29 [1.21-1.37]**

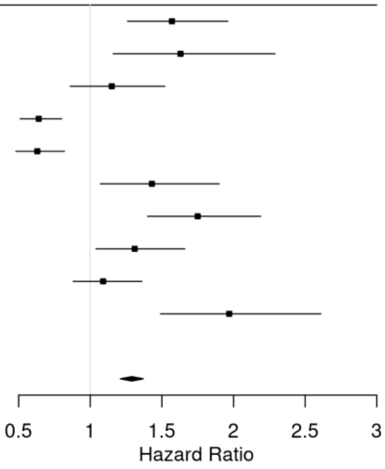

b)

2 Year Conditional Survival

Variable	P-value	HR [95% CI]
Age 65+	5.85e-04	1.55 [1.21-2.00]
Calcium 11+	0.525	1.16 [0.73-1.85]
LDH > 222	0.829	0.96 [0.67-1.38]
Early ASCT	7.78e-04	0.65 [0.50-0.83]
First-Line PI+IMID	0.014	0.67 [0.48-0.92]
Ch17 Abnormality	0.137	1.30 [0.92-1.82]
ISS Stage III	1.45e-05	1.76 [1.36-2.28]
1q Gain	2.49e-03	1.51 [1.16-1.97]
Monosomy 13	0.720	1.05 [0.82-1.34]
HR Translocation	6.25e-04	1.81 [1.29-2.55]
**Summary**		**1.25 [1.16-1.35]**

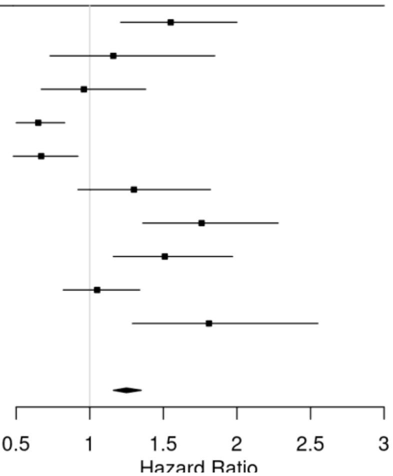

c)

3 Year Conditional Survival

Variable	P-value	HR [95% CI]
Age 65+	7.17e-03	1.48 [1.11-1.96]
Calcium 11+	0.865	1.05 [0.59-1.86]
LDH > 222	0.556	1.13 [0.76-1.68]
Early ASCT	7.45e-04	0.61 [0.46-0.81]
First-Line PI+IMID	0.394	0.66 [0.45-0.98]
Ch17 Abnormality	0.167	1.32 [0.89-1.95]
ISS Stage III	8.27e-05	1.80 [1.34-2.41]
1q Gain	9.77e-03	1.50 [1.10-2.05]
Monosomy 13	0.733	1.05 [0.79-1.39]
HR Translocation	0.037	1.55 [1.03-2.34]
**Summary**		**1.23 [1.13-1.34]**

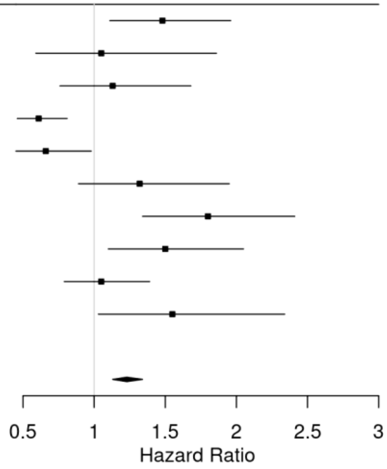

d)

5 Year Conditional Survival

Variable	P-value	HR [95% CI]
Age 65+	4.17e-04	1.95 [1.34-2.82]
Calcium 11+	0.909	1.05 [0.46-2.41]
LDH > 222	0.699	0.89 [0.48-1.63]
Early ASCT	0.051	0.69 [0.48-1.00]
First-Line PI+IMID	0.042	0.53 [0.29-0.98]
Ch17 Abnormality	0.620	1.16 [0.64-2.09]
ISS Stage III	0.054	1.52 [0.99-2.32]
1q Gain	0.152	1.38 [0.89-2.13]
Monosomy 13	0.417	0.86 [0.59-1.25]
HR Translocation	0.198	1.51 [0.8-2.85]
**Summary**		**1.20 [1.05-1.35]**

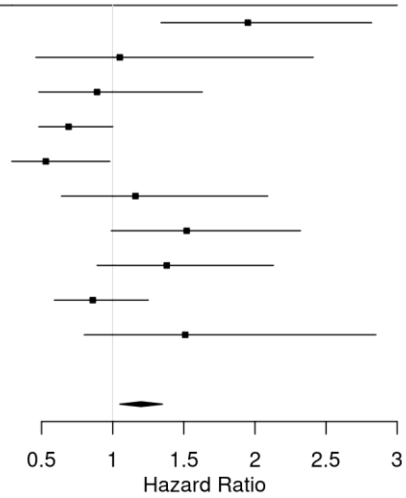

Supplemental Figure 1: Forest Plots of Multivariate Conditional Survival Models for patients surviving a) 1 year, b) 2 years, c) 3 years, and d) 5 years from diagnosis. *Abbreviations: ASCT: autologous stem cell transplantation, CI: confidence interval, HR: hazard ratio, IMiD: immunomodulatory drug, ISS: international staging system, LDH: lactate dehydrogenase, PI: proteasome inhibitor*
